# Novelty Detection using Deep Normative Modeling for IMU-Based Abnormal Movement Monitoring in Parkinson’s Disease and Autism Spectrum Disorders

**DOI:** 10.3390/s18103533

**Published:** 2018-10-19

**Authors:** Nastaran Mohammadian Rad, Twan van Laarhoven, Cesare Furlanello, Elena Marchiori

**Affiliations:** 1 Institute for Computing and Information Science, Radboud University, 6525EC Nijmegen, The Netherlands; mail@twanvl.nl (T.v.L.); elenam@cs.ru.nl (E.M.); 2 Department of Information Engineering and Computer Science, University of Trento, 38123 Trento, Italy; 3 Fondazione Bruno Kessler, 38123 Trento, Italy; furlan@fbk.eu; 4 Faculty of Management, Science and Technology, Open University of the Netherlands, 6419AT Heerlen, The Netherlands

**Keywords:** novelty detection, deep learning, normative modeling, denoising autoencoders, Parkinson’s disease, autism spectrum disorder, stereotypical motor movements, freezing of gait

## Abstract

Detecting and monitoring of abnormal movement behaviors in patients with Parkinson’s Disease (PD) and individuals with Autism Spectrum Disorders (ASD) are beneficial for adjusting care and medical treatment in order to improve the patient’s quality of life. Supervised methods commonly used in the literature need annotation of data, which is a time-consuming and costly process. In this paper, we propose deep normative modeling as a probabilistic novelty detection method, in which we model the distribution of normal human movements recorded by wearable sensors and try to detect abnormal movements in patients with PD and ASD in a novelty detection framework. In the proposed deep normative model, a movement disorder behavior is treated as an extreme of the normal range or, equivalently, as a deviation from the normal movements. Our experiments on three benchmark datasets indicate the effectiveness of the proposed method, which outperforms one-class SVM and the reconstruction-based novelty detection approaches. Our contribution opens the door toward modeling normal human movements during daily activities using wearable sensors and eventually real-time abnormal movement detection in neuro-developmental and neuro-degenerative disorders.

## 1. Introduction

Recent advances in wearable sensor technology, and more specifically Inertial Measurement Unit (IMU) sensors, have provided an effective platform for remote monitoring of patients with motor malfunctions such as Parkinson’s Disease (PD) [1] and Autism Spectrum Disorder (ASD) [2]. IMUs contain built-in accelerometers, gyroscopes and magnetometer sensors allowing one to measure the angular velocity and linear acceleration of body parts during movement. IMUs—due to their small size, high portability and light weight—have become some of the most popular devices in human action recognition and abnormal movement detection. Especially in psychiatric clinical studies, IMUs not only provide the possibility to measure the kinetic symptoms and phenotypes automatically, but also, they enable caregivers to follow up on the progress of diseases and the quality of interventions more frequently than the current clinical practices [3,4].

ASD and PD are respectively neuro-developmental and neuro-degenerative disorders, each with different symptoms involving atypical motor movements. PD affects the motor system causing motor symptoms such as tremors, bradykinesia (slowness), Freezing of Gait (FOG), and muscle rigidity [5]. These abnormal motor defects significantly impair patients’ quality of life. Among them, FOG increases the risk of falling generally in elderly PD patients. ASD has also some specific motor behavior symptoms such as Stereotypical Motor Movements (SMMs) [6]. SMMs are the major group of abnormal repetitive behaviors, e.g., hand flapping and body rocking, in children with ASD. These atypical motor movements decrease the performance of children while learning new skills or using learned skills. In addition, since these behaviors are socially abnormal, they cause difficulties in social interaction with other peers. In the case of severity, SMMs can even lead to self-injury behaviors.

Recently, many research studies have focused on detecting abnormal movements in patients with mental or brain disorders, such as SMMs in children with ASD and FOG in PD patients, using wearable sensors [3,7,8,9]. These studies have mainly concentrated on applying supervised machine learning algorithms to classify samples of abnormal movements from normal ones. There are three main challenges in applying supervised approaches for abnormal movement detection: (i) they generally rely on the availability of labeled data while, especially in this context, data labeling is an expensive, time-consuming and subjective task [10,11,12], as it needs full monitoring of subjects during the data collection phase; (ii) severe class distribution skewness, where samples in the normal class severely out-represent abnormal samples in recorded data from patients with ASD and PD [13,14]; this fact makes the classification techniques sub-optimal for these applications; (iii) the heterogeneity of non-stationary patterns in normal and abnormal movements that makes the task of finding a separating hyper-cube in classification scenarios even more cumbersome [15].

As an alternative for supervised approaches, novelty detection provides all the ingredients needed for tackling the aforementioned challenges in an unsupervised fashion. In general, novelty detection is defined as the task of learning the overall characteristics of available normal samples in the training phase and then using these characteristics to recognize novel samples that differ in some respects from the normal samples at test time [11,16]. Based on this definition, novelty detection approaches naturally need only samples of the normal class in the training phase; hence, they do not need labeled data and are immunized against highly imbalanced class distributions. More importantly, adopting a probabilistic policy in novelty detection enables us to estimate the generative probability density function of the normal data, which can cover a wide and heterogeneous spectrum of normal samples. These advantages made novelty detection techniques very successful in many applications ranging from fraud detection [17,18], medical diagnosis [19,20,21], fault detection [22,23], to anomaly and outlier detection in sensor networks [24,25], video surveillance [26,27] and text mining [28,29].

In this paper, we adopt a probabilistic novelty detection approach based on normative modeling [30] in order to, first, model heterogeneous normal movements in PD and ASD and, second, to use the resulting model in a novelty detection paradigm to detect FOGs and SMMs in respectively PD and ASD patients. To this end, by assuming a multivariate normal distribution on the collected accelerometer signals of normal movements and exploiting the underlying principles of probabilistic deep neural networks [31], we extend the applications of normative modeling to unimodal datasets. In general, a normative model is constructed in the training phase by estimating a mapping function between two different data modalities, e.g., behavioral covariates and biological measurements. In some applications, such as ours, only one modality of data is available. To overcome this barrier, we use the denoising autoencoder (DAE) to reconstruct the original IMU signals of normal movements from their noisy versions. In fact, the model implicitly learns the distribution of the normal movements. Using dropout layers in the DAE architecture enables us to estimate also the variance of predictions (which is necessary for normative modeling) in addition to mean predictions. We compare the proposed method with state-of-the-art supervised approaches, as well as classic one-class classification and reconstruction-based novelty detection. Our experimental results on three benchmark datasets illustrate that the proposed method provides a reasonably close performance to its supervised counterparts, whilst yielding the best performance among other competing novelty detection approaches on three benchmark datasets.

The rest of the paper is organized as follows. Section 2 briefly reviews the state-of-the-art of novelty detection techniques for abnormal movement detection. Section 3 presents our proposed unsupervised novelty detection approach based on normative modeling. The experimental materials and the procedures are also described in this section. Section 4 compares our experimental results versus other novelty detection and supervised methods. In Section 5, we discuss the advantages and limitations of the proposed method and state the possible future directions.

## 2. Related Works

Recent studies on automatic SMM and FOG detection using wearable sensors have mainly focused on applying supervised machine learning and deep learning approaches, such as Convolutional Neural Network (CNN) and Long Short-Term Memory (LSTM), to distinguish between the normal and abnormal movements [9,32,33,34,35,36,37]. These methods are based on extracting or learning a set of robust features from the original signals and then applying the supervised algorithms for abnormal movement detection. The main drawback of these approaches, however, is their need for labeled data. To overcome this problem, few studies have recently focused on using novelty detection methods [38,39]. In a FOG detection application, Cola et al. [38] used a distance-based novelty detection method on accelerometer signals to detect abnormal gait patterns. Their proposed method consists of extracting a set of hand-crafted features and then applying a K-Nearest Neighbor (KNN) method. The KNN approach assumes that normal gait samples are located at the close distance from each other. Thus, a sample is determined as an abnormal sample if it is located far from its neighbors. Their proposed method achieved on average 80% accuracy for detecting abnormal gait samples. Despite the reported high accuracy rate, the high computational complexity of KNN at the test time severely limits its application in real-time applications. Elsewhere, Nguyen et al. [39] proposed a probabilistic novelty detection method for abnormal gait recognition in musculoskeletal disorders using Microsoft Kinect^®^ sensors. Their method was based on training a Hidden Markov Model (HMM) to model the transition of human posture states in a gait cycle. Then, to distinguish between the normal gait samples from the abnormal ones, a threshold was defined based on the mean and standard deviation of the estimated log-likelihood on normal gait samples.

Recently, deep learning approaches were also used for novelty detection applications. Erfani et al. [40] proposed a hybrid model of an autoencoder and one-class SVM for detecting anomalies in high-dimensional and large-scale datasets including a daily activity dataset. A set of learned features by autoencoders was fed to a one-class SVM in order to detect the abnormal samples. Their experimental results showed the superiority of using one-class SVM in the learned latent space rather than the original raw signal space. Autoencoders are also widely used for detecting abnormal patterns in medical images through the reconstruction error between the output of the model and the actual input [41,42]. Novelty detection based on reconstruction error was also used by Khan and Taati [43] for fall detection using wearable sensors. The proposed approach was based on using a channel-wise ensemble of autoencoders for data reconstruction and setting a threshold on the reconstruction error to distinguish the falling instances.

## 3. Methods

In the context of abnormal movement detection using wearable sensors, novelty detection is defined as detecting atypical movements in the test phase while only normal movements are available in the training phase. In this study, we consider a probabilistic novelty detection approach consisting of the following three steps: (1) learning the distribution of normal movements using a probabilistic denoising autoencoder; (2) quantifying the deviation of each test sample from the distribution of normal movements, the so-called Normative Probability Map (NPM), in the normative modeling framework; (3) computing the degree of novelty of each test sample by fitting a generalized extreme value distribution on summary statistics of its NPM.

**Figure 1:**
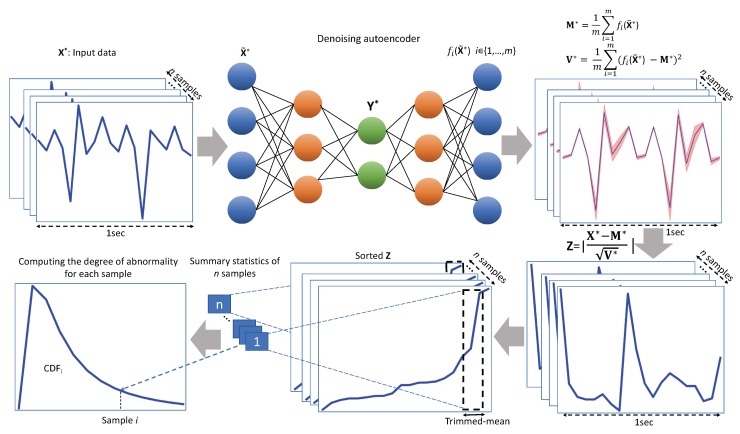
The proposed method for the abnormal movement detection in the test time.

We formalize these three steps in the next 3 subsections. Figure 1 also shows the proposed method.

In this text, we use boldface capital letters to represent matrices, boldface lowercase letters to represent vectors and italic lowercase letters to represent scalars.

### 3.1. Learning the Distribution of Normal Movements via the Denoising Autoencoder

As stated in the previous section, our method starts by modeling the normal movements. To do this, we use convolutional neural networks, which are the state-of-the-art for activity recognition and movement monitoring using wearable sensors. In particular, we train a Denoising Autoencoder (DAE), which is a type of (autoencoding) neural network that aims to reconstruct (denoise) its inputs from noisy samples.

More formally, given a training set $X_{N}\in R^{n_{N}\times p}$ consisting of $n_{N}$ samples of normal movements drawn from a distribution $P_{N}$ of normal movements, a trained DAE is a function $f_{N}$ that has the property that $f_{N}\left(X+\epsilon \right)\approx X$ for $X\in X_{N}$. Given sufficient training data, the network generalizes to reconstruct any $X\in P_{N}$.

How well the autoencoder is able to denoise its input is proportional to how well that input matches the distribution of the training data, in our case how well the input matches a normal movement. Hence, we can use the distance between the reconstruction of DAE and the true sample, the reconstruction error, as a measure of the likelihood $P_{N}\left(X\right)$ of the sample.

However, the neural network only produces a point estimate, that is a single possible reconstruction given a noisy input. For some features or samples, this prediction might be very accurate, while others can be much harder to reconstruct. The reconstruction error does not take this prediction uncertainty into account.

To use the prediction uncertainty properly, we use the NPM, introduced in [30]. The original NPM method used Gaussian processes to model the normal data, which also provide a variance as a measure of uncertainty. To calculate the variance of the predictions in our denoising autoencoder setup, we instead use dropout [31], to make the network nondeterministic. As shown by Gal and Ghahramani [31], using Monte Carlo sampling by applying dropout at test time provides an approximation of the posterior $P_{N}\left(\theta |X\right)$. After drawing *m* samples from the predictive distribution, we can calculate their empirical mean and variance,

(1a) $$\begin{array}{cc} M^{*}= & \frac{1}{m}\sum_{i=1}^{m}f_{i}\left({\tilde{X}}^{*}\right) \end{array}$$

(1b) $$\begin{array}{cc} V^{*}= & \frac{1}{m}\sum_{i=1}^{m}{\left(f_{i}\left({\tilde{X}}^{*}\right)-M^{*}\right)}^{2}. \end{array}$$

Here, $f_{i}$ indicates the different variations of the autoencoder network, which are formed by applying dropout.

### 3.2. Quantifying the Deviation from $P_{N}$

In this study, we adapt the normative modeling framework in order to quantify the deviation of each newly-seen test sample from the distribution of normal movements $P_{N}$. In this framework, the mean and variance of the reconstruction are used to compute an NPM,

(2) $$\begin{array}{c} Z=\frac{X^{*}-M^{*}}{\sqrt{V^{*}}}. \end{array}$$

These NPM scores are in fact z-scores, quantifying the deviation of samples in $X^{*}$ from a reconstructed normal sample under $P_{N}$, in units of standard deviation of the predictive distribution [44]. It combines two sources of information: (1) the prediction error (difference between the true and expected predicted responses) and (2) the predictive variance of the test points.

### 3.3. Computing the Degree of Novelty

The NPM score of each test sample is a *p*-dimensional multivariate measure of deviation. It quantifies the deviation for each of the *p* responses of a test sample. In order to summarize these deviations into a degree of abnormality, we follow [30] and employ the Generalized Extreme Value Distribution (GEVD) [45,46] to model the samples in the extreme tails of $P_{N}$ (see Appendix A for more details). In fact, we consider that abnormal motor movements may occur as an extreme deviation from a normal pattern. As in [30], we adopt a “block maxima” approach where we compute the 90% trimmed mean of the top 1% values in *Z* of each sample in order to summarize the deviations as a single number. Then, to make probabilistic subject-level inferences about these deviations, we fit a GEVD on the resulting summary statistics. The cumulative density function of the resulting GEVD at a given test sample then can be used as the probability of each sample being an abnormal sample [47].

### 3.4. Experimental Materials

We compare the performance of the proposed probabilistic novelty detection approach with reconstruction-based novelty detection [16,40], one-class Support Vector Machine (SVM) [48] and supervised deep learning approaches on two datasets: (i) an SMM dataset collected in a longitudinal study from children with ASD [3] (the SMM dataset and the full description of the data are publicly available at https://bitbucket.org/mhealthresearchgroup/stereotypypublicdataset-sourcecodes/downloads) and (ii) the Daphnet Freezing of Gait dataset collected from PD patients [49]. In the following, we detail the datasets and the preprocessing steps.

#### 3.4.1. Datasets

The SMM dataset contains accelerometer recordings from 6 individuals with ASD who had a significant score on the RSB-R [50] for body rocking and hand flapping. The data were collected in two sessions from the same participants, here referred to as SMM-1 and SMM-2. During data collection, participants wore three 3-axis accelerometer sensors on their torso, right wrist and left wrist. Data for SMM-1 were collected using MIT sensors at a 60-Hz frequency rate. SMM-2 was recorded using Wockets sensors with a sampling frequency of 90 Hz. The recordings were annotated offline by an expert using the recorded video. To equalize the sampling rate of two recordings, the signal in SMM-1 was resampled to 90 Hz using a linear interpolation. Then, the cutoff high-pass filter with 0.1 Hz was applied to remove the DC components in the signal. Finally, the signal was segmented to 1 s-long intervals with 0.87% overlap between consecutive windows.

The data in the Daphnet Freezing of Gait dataset [49], here referred to as FOG, were collected from 10 PD patients at a 64-Hz frequency rate while participants wore three 3-axis accelerometer sensors on their shank, thigh and belt. During the experiment, participants were instructed to perform walking tasks. The whole experiment was recorded with a digital video camera. Then, two physiotherapists annotated the FOG episodes using the video recordings. Following the preprocessing stage in [8], we first downsampled the accelerometer data to 32 Hz. The data were then segmented into 1 s-long intervals using a sliding window. The sliding window was moved along the time dimension with 10 time-steps to make overlaps between consecutive windows.

In the segmentation phase, segments with normal movement samples were selected to train the model. Other partial normal segments were removed from the training data. Table 1 summarizes the number of normal and abnormal samples for each subject in the SMM and FOG datasets. The difference in the number of samples in the abnormal and normal classes represents the unbalanced nature of data where in the SMM-1 and SMM-2 datasets, 31% and 23% of samples are in the SMM class, and in the FOG dataset, 11% of samples are in the FOG class.

#### 3.4.2. Network Architectures

Considering their different rhythmic characteristics, we used different network architectures for the FOG and SMMs datasets. We adopted the CNN architecture that was proposed by Hammerla et al. [8] for the FOG dataset and the CNN architecture proposed by Rad et al. [7] for the SMM datasets. In the following, we detail how these architectures are manipulated to serve our purpose explained in Section 3 (the Keras library [51] is used to implement DAE and CNN architectures).

**Figure 2:**
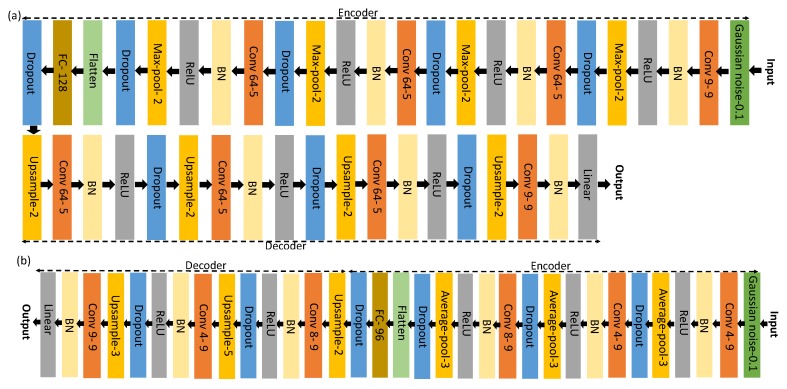
The architecture of convolutional denoising autoencoder for (**a**) the FOG dataset and (**b**) the SMM dataset. Each colored box represents one layer. The type and configuration of each layer are shown inside each box. For example, Conv 64-5 denotes a convolutional layer with 64 filters and 5 kernel size.

- DAE architecture for the FOG dataset: The original CNN architecture in Hammerla et al. [8] was used for encoding the signal into a lower dimensional representation. This architecture contains four convolutional layers alternating convolution, batch normalization, Rectified Linear Units (ReLU) and max-pooling layers to map the large input space to a lower dimensional feature space. A fully-connected layer is then stacked on top of the fourth convolution layer to form the encoder. We concatenate a mirror reversal of the encoder network to the last encoder layer in order to reconstruct the input signal in a DAE architecture. In the decoding part, we replace max-pooling layers with up-sampling layers. In order to capture the model uncertainty, we placed a dropout layer before every weight layer [31]. The resulting architecture is shown in Figure 2a.
- DAE architecture for SMM datasets: Similar to [7], the encoder architecture consists of three convolutional layers, which alternates convolution, batch normalization, ReLUs and average-pooling layers to transform the raw feature space into a lower dimensional set of features. A fully-connected layer is then stacked on top of the third convolution layer. The resulting latent vector is then decoded in the decoder to reconstruct the input signal. Similar to the DAE architecture for the FOG dataset, the architecture of the decoder network is a mirror reversal of the encoder, and dropout layers are used before every weight layer. The architecture and the configuration of each layer are depicted in Figure 2b.

#### 3.4.3. Experimental Setups and Evaluation

We conducted four experiments to evaluate the performance of the proposed method against three competing approaches:

- Experiment 1, normative modeling: We followed the proposed procedure explained in Section 3, using the DAE architectures described in Section 3.4.2 for learning the distribution of the normal movements on the SMM-1, SMM-2 and FOG datasets. In this setting, models are trained in an unsupervised manner and only on the samples of normal movements. For training the DAEs, we used the RMSprop optimizer to minimize the mean squared error loss function. To compute $M^{*}$ and $V^{*}$, we drew m=50 MC samples from DAE predictions, and the mean and variance across these 50 MC samples are used to compute the $M^{*}$ and $V^{*}$ matrices. In all experiments, we fix the dropout level to 0.1. Later in order to investigate the effect of the dropout level on the performance of the proposed novelty detection approach, we repeat this experiment for different dropout probability levels δ={0.1,0.2,0.3,0.4,0.5} and compare the results.
- Experiment 2, reconstruction-based: The goal of this experiment is to assess the effect of incorporating prediction uncertainties, i.e., $V^{*}$, on the performance of the novelty detection system. All the experimental settings in this experiment are similar to Experiment 1, except for computing the NPMs, where we use $Z=X^{*}-M^{*}$ instead of Equation (2). Since in this setting, only the reconstruction error is used to construct a model of normal movements, we refer to this experiment as “reconstruction-based”.
- Experiment 3, one-class SVM: The goal in this experiment is to compare the proposed method for novelty detection with one-class classification. To this end, we train a one-class SVM model in a novelty detection setting [16,40,52,53]. One-class SVM fits a hyper-sphere decision boundary on a nonlinearly-transformed feature space to include the majority of samples in the normal class and detects anomalies as deviations from the learned decision boundary. In this experiment in a similar setting used by Erfani et al. [40], we use the learned reduced-rank latent space via the DAE model, i.e., $Y_{N}\in R^{n_{N}\times q}$, to train a one-class SVM model. We use this model later to distinguish the normal and abnormal movements on the samples. For the one-class SVM, we employed the implementation available in the scikit-learn [54] package. We used the Radial Basis Function (RBF) kernel with default hyperparameters, where ν=0.5 and $\gamma =\frac{1}{q}$ (Considering our assumption that only normal movement samples are available during the training phase, fine-tuning these hyperparameters is not possible. See Section 5.2 for the discussion.).
- Experiment 4, supervised: To compare the performance of the proposed unsupervised novelty detection technique with supervised classification, we used the CNN architecture proposed in Hammerla et al. [8] and Rad et al. [7] on the FOG and SMM datasets, respectively, in a fully-supervised scenario.

Note that since the samples for Subjects 4 and 10 in the FOG dataset only contain normal movements (see Table 1), it is not possible to evaluate the benchmark approaches on these two subjects in Experiments 1–3. Thus, in an extra setting, we repeat Experiments 1–3 when only these two subjects are used in the training phase. This setting is even more close to the reality as only subjects with normal movements are available during the training phase (in this case, there is no need for the additional preprocessing procedure to select the normal segments).

In all experiments, the leave-one-subject-out cross-validation is used for the model evaluation, and the area under the receiver operating characteristic curve (ROC), i.e., AUC, is computed as the performance measure. The whole experimental procedures are repeated 5 times to report the standard deviation over the mean AUC performances.

## 4. Results

Table 2 summarizes single-subject and average AUC measures for the four experiments that were described in Section 3.4.3 on the FOG, SMM-1, and SMM-2 datasets.

On the FOG dataset, we observed a large variance of results across subjects. In particular, the normative modeling and reconstruction-based methods achieved a much lower AUC performance on Subjects 6 and 8 than on the other subjects. These two subjects were the only females in the dataset exhibiting atypical movement behavior (see Table 1). A potential explanation for the lower performance is that, when training on mainly male subjects, novelty detection models, which use the reconstruction error, are unable to reconstruct normal female movement behavior correctly. On the SMM datasets, the performance was more similar across subjects, notably on the SMM-1 dataset. This could be due to the controlled setting used to collect data: while wearing the sensors, participants were observed in the lab, sitting in a comfortable chair with a familiar teacher [55]. Results on the FOG dataset also indicated the presence of possible biases due to the limited size of the data from normal subjects (see also the results reported in Section 4.4). The public availability of larger datasets would allow a more thorough assessment of the methods for abnormal movement detection in PD and ASD, which would be highly beneficial to advance patient care and research. The results are further investigated in the following sections.

### 4.1. Normative Modeling Outperforms Reconstruction-Based and One-Class SVM in Novelty Detection

The comparison between results achieved by our normative modeling method and its reconstruction-based variant indicate the beneficial effect of incorporating the uncertainty of the predictions in the NPM scores for the FOG dataset. In this context, for all subjects, the normative modeling method outperformed the reconstruction-based one. On this dataset, normative modeling also outperformed one-class SVM on all except one subject. These results illustrate the effectiveness of normative modeling method for detecting movement disorder behavior in PD patients.

On the SMM-1 and SMM-2 datasets, normative modeling and reconstruction-based modeling methods achieved similar performance. This indicates that the uncertainty of the prediction did not significantly affect the ranking of the samples obtained using the reconstruction-error scores. On this dataset, the performance of one-class SVM was not very satisfactory. This result can be explained by the fact that one-class SVM does not rely on the properties of the distribution of the training data; rather, it fits a decision boundary on a nonlinearly-transformed feature space to include the majority of samples in the normal class and detects anomalies as samples falling outside the learned decision boundary. Therefore, the performance of this method is highly dependent on selecting proper parameters to control the size of the boundary.

### 4.2. Novelty Detection Methods vs. Supervised Learning Methods

Our experimental results in Table 2 demonstrate that our normative modeling method provided a reasonably close performance to its supervised counterpart on the SMM-1 dataset and a relatively close performance to the supervised method for the SMM-2 and FOG datasets. In particular, on the FOG dataset, in two cases (Subjects 1 and 5), the normative modeling method outperformed the supervised method (with a 7% and 3% improvement, respectively). Furthermore, on the SMM-1 dataset, the reconstruction-based method outperformed the supervised method in two cases, Subjects 2 and 5, with a 5% and 4% improvement, respectively.

**Figure 3:**
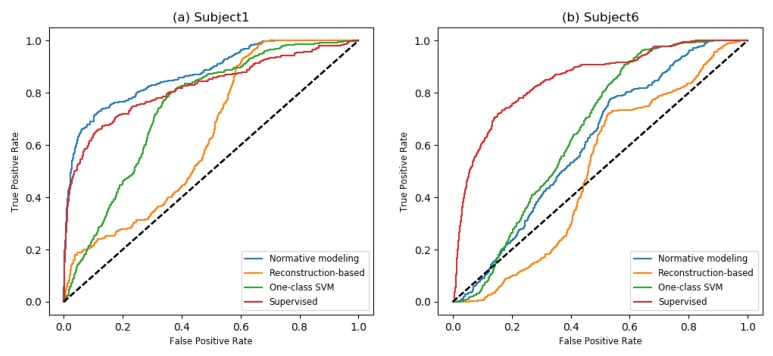
ROC curves corresponding to the reported AUCs for Subjects 1 and 6 (**a**,**b**) of the FOG dataset in Table 2.

To get a summarized demonstration of the performance of different novelty detection methods, we consider the best and the worst normative modeling results on the FOG dataset, i.e., Subjects 1 and 6. ROC curves for these subjects are depicted in Figure 3. In Figure 3a, we can see that both the reconstruction-based and normative modeling methods were able to identify the most normal (negative) data for Subject 1 correctly. However, the reconstruction-based approach was not able to find the most likely abnormal movement (positive) samples. Figure 3b shows the results for Subject 6. Here, around 1/4 of the samples were clearly identified as normal by most methods; however, the other samples could not be distinguished. In the normative modeling method, both positive and negative samples were assigned a high likelihood of being abnormal, perhaps because the normal movements for this subject differed too much from those in the training data.

Since the datasets presented in this paper are highly skewed, especially the FOG dataset, in addition to AUC, we also evaluated the performance of the methods using the Area Under the PRC curve (AUPR) [56]. Compared to AUC, the AUPR score places more weight on the highly ranked predictions by each method. As is shown in Table 3, on the FOG dataset, the normative modeling method achieved a higher average AUPR than other novelty detection methods. For some subjects, in particular Subject 6, all of the novelty detection methods showed low performances. We believe this is because this subject was too different from the training data, and hence, none of the methods found clear FOG signals, which can also be seen in Figure 3b. On the SMM datasets, normative modeling and reconstruction-based methods achieved comparable performance in terms of AUPR, while both clearly outperformed one-class SVMs. The AUPR scores for the autoencoder-based methods were quite high on this dataset, which indicates that they were able to find clear instances of SMM behaviors in all subjects correctly.

### 4.3. Effect of Dropout Level

**Figure 4:**
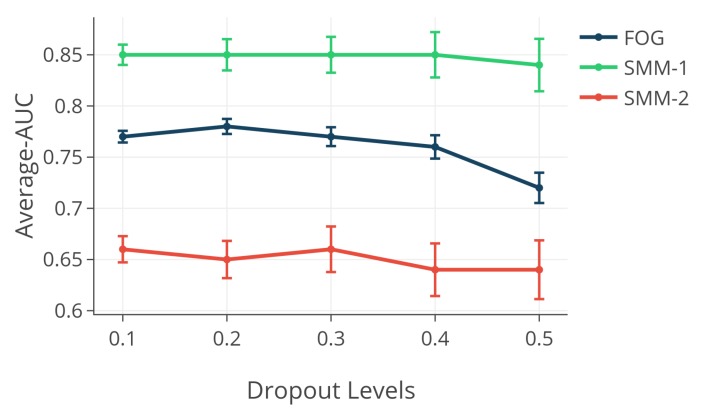
The effect of different dropout probabilities on the performance of the normative modeling method.

Figure 4 depicts the effect of different dropout probabilities on the performance of the normative modeling method on the SMM-1, SMM-2 and FOG datasets with the leave-one-subject-out scheme. As is shown in Figure 4, using the different dropout probabilities had a negligible effect on the performance of the normative modeling method for the SMM and FOG datasets. Thus, a value between 0.1 and 0.4 can be used as the dropout probability level without a significant drop in the performance.

### 4.4. Training Only on Normal Subjects

It is interesting to investigate how our novelty detection methods perform when only data from subjects without atypical movement behavior are present in the training set. In this setting, the expert interaction and preprocessing time were reduced. Therefore, in this experiment, we trained the considered novelty detection models only on two normal subjects, i.e., Subjects 4 and 10 in the FOG dataset (see Table 1). Results of this experiment are shown in Table 4. As expected, there is a drop in the average performance compared to the results of Experiment 1 (see the FOG results in Table 2), which is likely due to the limited training data with just two subjects. Interestingly, in this setting, the normative modeling method improved its performance on Subject 2 (0.92 average AUC), showing that the normal movement behavior of this subject was closer to that of Subjects 4 and 10 than to that of the other subjects. Overall, the results of normative modeling and reconstruction-based methods decreased when using less data, while the results of one-class SVM did not change significantly, indicating that the latter method is incapable of exploiting information from more subjects.

## 5. Discussion

### 5.1. Estimating the Prediction Uncertainty: Deep Learning vs. Gaussian Processes

Considering our multi-variate Gaussian assumption on the distribution of the IMU signal of normal movements, Multi-task Gaussian Process Regression (MTGPR) [57] seemed to be a natural choice for estimating the structured prediction uncertainty in normative modeling. However, MTGPR comes with extra computational overheads in time and space ($O(n_{N}^{3}p^{3})$ and $O(n_{N}^{2}p^{2})$) when computing the inverse cross-covariance matrices in the optimization and prediction phases. This problem is even more pronounced when dealing with multi-subject IMU-based abnormal movement detection when generally $n_{N}$ is in order of $10^{5}$ to $10^{6}$. Despite extensive studies to reduce these computational barriers [21,58,59,60], the overall efficiency of the proposed approaches remained far below the minimum requirements in our target applications. To overcome this problem, in this study, we proposed to replace the MTGPR with a probabilistic DAE architecture for estimating the prediction uncertainties in the normative modeling framework. As supported by our experimental results, the estimated prediction uncertainties via DAE edged the novelty detection performance in comparison with the reconstruction-based approach. Our contribution facilitates the application of normative modeling on the large datasets (with large $n_{N}$ or *p*) in the big-data era.

### 5.2. Normative Modeling vs. One-Class Classification

One-class classification [61] and more specifically one-class SVM is a common choice for solving novelty detection problems [16,52,53]. It is shown that one-class SVMs achieve poor performance on high-dimensional datasets, while a combination of a feature extraction method such as deep belief networks with one-class SVM enhances the performance of such novelty detection methods [40]. However, the prediction performance of one-class SVM is highly sensitive to its hyperparameters (e.g., in the case of RBF kernel ν and γ), especially on noisy data. This fact is well demonstrated in our experiments, where one-class SVM performed better when trained only on normal subjects; data, i.e., less noisy data (compare the results in Table 2 and Table 4). Therefore, fine-tuning of one-class SVM hyperparameters is necessary; however, this is only possible if we have access to the labeled validation data during the model selection phase. This limitation leaves the only option of using default parameters when dealing with non-labeled data, which results in sub-optimal performances. The proposed deep normative modeling approach for novelty detection overcomes this barrier, as our experiments on three benchmark datasets show that its only hyperparameter, i.e., the dropout level, can be set to 0.1–0.4 without a significant drop in the prediction performance.

### 5.3. Toward Modeling Human Normal Daily Movements Using Wearable Sensors

The majority of research studies in detecting human pathological movements using wearable sensors is mainly focused on classifying the normal movements from the abnormal ones. These approaches suffer from major deficits in supervised learning such as the lack of labeled samples and lack of generalization to newly-unseen movements. A possible turn around is to define the problem in an unsupervised framework and try to assemble a probabilistic model of human normal daily movements. If successful, then in, for example, a novelty detection scenario, any large deviation from this model can be considered as an abnormal movement for the diagnosis and treatment of patients with motor deficiencies. Of course, learning a realistic representation of all possible human movements is very challenging due to the large set of possible movements, inter- and intra-subject heterogeneity and the prevalence of noisy samples. The proposed deep normative modeling method provides an early, but effective step toward this direction as it provides all the needed ingredients for modeling heterogeneous normal human movements in an unsupervised fashion.

### 5.4. Limitations and Future Work

Using DAE for learning $P_{N}$ limits the application of the proposed method only to distance-based novelty detection approaches in the original and latent space; hence, it is not applicable in the density-based novelty detection [41]. This is because the DAE model is by nature unable to determine the density of normal data in the latent space. To address this problem, one possible future direction is to use generative alternative models instead of DAE such as variational autoencoders [62], adversarial autoencoders [63] or generative adversarial networks [64]. Another future direction is to use the proposed framework for implementing a real-time mobile application for abnormal movement detection. The proposed DAE-based normative modeling approach, unlike its MTGPR-based alternatives, does not need to store huge inverse covariance matrices at the test time. Adding to this the low computational complexity of DAE at the prediction phase (just matrix multiplications and summations) and high potential for parallel programming (for computing MC repetitions), the proposed method offers a very well-suited approach for online mobile novelty detection applications.

## 6. Conclusions

In this study, we addressed the problem of automatic abnormal movement detection in ASD and PD patients in a novelty detection framework. In the normative modeling framework, we used a convolutional denoising autoencoder to learn the distribution of the normal human movements from the accelerometer signals. We showed how the normative modeling framework can be employed to quantify the deviation of each unseen sample from the normal movement samples. We demonstrated empirically that our proposed method outperforms two other baseline novelty detection methods on the SMM and FOG datasets. Our method: (i) overcomes the high computational complexities of estimating the prediction uncertainties in multi-task normative modeling, thus facilitating its application to large datasets in the big-data era; (ii) unlike the common one-class classification setting, our method relaxes the need for having access to the labeled validation data during the model selection phase; and more importantly, (iii) our method provides the first step toward modeling human normal daily movements using wearable sensors. The proposed approach gathers all the required ingredients for implementing a real-time mobile application for abnormal movement detection in the future.

## Figures and Tables

**Table 1:** The class distribution of normal and abnormal samples and the gender of patients in three datasets.

Data	Subject	#Normal	#Abnormal	All	Abnormal/All	Gender
**FOG**	**Sub1**	5714	334	6048	0.06	M
	**Sub2**	3918	578	4496	0.13	M
	**Sub3**	5488	912	6400	0.14	M
	**Sub4**	6592	0	6592	0	M
	**Sub5**	5139	1517	6656	0.23	M
	**Sub6**	5917	419	6336	0.07	F
	**Sub7**	4858	262	5120	0.05	M
	**Sub8**	1812	620	2432	0.25	F
	**Sub9**	4673	863	5536	0.16	M
	**Sub10**	7104	0	7104	0	F
	**Total**	50,482	6238	56,720	0.11	-
**SMM-1**	**Sub1**	21,292	5663	26,955	0.21	M
	**Sub2**	12,763	4372	17,135	0.26	M
	**Sub3**	31,780	2855	34,635	0.08	M
	**Sub4**	10,571	10,243	20,814	0.49	M
	**Sub5**	17,782	6173	23,955	0.26	M
	**Sub6**	12,207	17,725	29,932	0.59	M
	**Total**	106,395	47,031	153,426	0.31	-
**SMM-2**	**Sub1**	18,729	11,656	30,385	0.38	M
	**Sub2**	22,611	4804	27,415	0.18	M
	**Sub3**	40,557	268	40,825	0.01	M
	**Sub4**	38,796	8176	46,972	0.17	M
	**Sub5**	22,896	6728	29,624	0.23	M
	**Sub6**	2375	11,178	13,553	0.82	M
	**Total**	145,964	42,810	188,774	0.23	-

**Table 2:** The average of AUC results for novelty detection using normative modeling, reconstruction-based and one-class SVM on three benchmark datasets.

Dataset	Subject	Normative	Reconstruction	1C-SVM	Supervised
**FOG**	**Sub1**	0.87±0.01	0.64±0.00	0.73±0.02	0.80±0.02
	**Sub2**	0.80±0.03	0.80±0.00	0.54±0.03	0.95±0.01
	**Sub3**	0.87±0.01	0.77±0.00	0.43±0.06	0.90±0.02
	**Sub5**	0.83±0.01	0.70±0.00	0.60±0.02	0.80±0.03
	**Sub6**	0.60±0.03	0.50±0.00	0.70±0.05	0.80±0.04
	**Sub7**	0.79±0.01	0.66±0.00	0.67±0.02	0.92±0.01
	**Sub8**	0.64±0.01	0.48±0.00	0.70±0.02	0.65±0.03
	**Sub9**	0.77±0.02	0.51±0.00	0.62±0.05	0.94±0.01
	**Mean**	0.77 ± 0.01	0.63±0.00	0.62±0.01	0.84±0.01
**SMM-1**	**Sub1**	0.86±0.01	0.71±0.00	0.32±0.01	0.89±0.01
	**Sub2**	0.85±0.01	0.88±0.00	0.23±0.03	0.83±0.02
	**Sub3**	0.87±0.02	0.91±0.00	0.22±0.03	0.93±0.01
	**Sub4**	0.88±0.03	0.81±0.01	0.31±0.04	0.95±0.00
	**Sub5**	0.76±0.03	0.87±0.01	0.26±0.01	0.83±0.03
	**Sub6**	0.88±0.03	0.82±0.00	0.30±0.02	0.93±0.01
	**Mean**	0.85 ± 0.01	0.83±0.00	0.28±0.01	0.89±0.01
**SMM-2**	**Sub1**	0.69±0.05	0.76±0.01	0.37±0.04	0.79±0.07
	**Sub2**	0.61±0.04	0.58±0.00	0.46±0.02	0.53±0.03
	**Sub3**	0.62±0.02	0.63±0.02	0.42±0.02	0.63±0.05
	**Sub4**	0.74±0.09	0.42±0.02	0.49±0.05	0.88±0.06
	**Sub5**	0.65±0.09	0.77±0.03	0.45±0.03	0.78±0.04
	**Sub6**	0.65±0.03	0.73±0.02	0.44±0.02	0.74±0.11
	**Mean**	0.66 ± 0.02	0.65±0.01	0.44±0.01	0.73±0.03

**Table 3:** The average AUPR for novelty detection using normative modeling, reconstruction-based and one-class-SVM on three benchmark datasets.

Dataset	Subject	Normative	Reconstruction	1C-SVM	Supervised
**FOG**	**Sub1**	0.48±0.02	0.11±0.00	0.11±0.02	0.38±0.02
	**Sub2**	0.32±0.03	0.51±0.01	0.13±0.01	0.79±0.03
	**Sub3**	0.51±0.01	0.35±0.00	0.12±0.02	0.55±0.02
	**Sub5**	0.54±0.01	0.36±0.00	0.28±0.02	0.59±0.03
	**Sub6**	0.08±0.01	0.06±0.00	0.10±0.02	0.33±0.06
	**Sub7**	0.22±0.01	0.09±0.00	0.09±0.01	0.49±0.04
	**Sub8**	0.38±0.01	0.26±0.00	0.43±0.05	0.34±0.04
	**Sub9**	0.42±0.03	0.19±0.00	0.21±0.05	0.71±0.05
	**Mean**	0.37 ± 0.01	0.24±0.00	0.18±0.01	0.52±0.01
**SMM-1**	**Sub1**	0.69±0.01	0.56±0.00	0.15±0.00	0.76±0.02
	**Sub2**	0.63±0.02	0.76±0.00	0.17±0.01	0.72±0.03
	**Sub3**	0.57±0.03	0.60±0.01	0.05±0.00	0.70±0.04
	**Sub4**	0.86±0.02	0.78±0.01	0.39±0.02	0.93±0.00
	**Sub5**	0.50±0.05	0.75±0.01	0.18±0.00	0.67±0.04
	**Sub6**	0.90±0.02	0.81±0.01	0.47±0.01	0.95±0.01
	**Mean**	0.69±0.01	0.71 ± 0.00	0.23±0.00	0.79±0.01
**SMM-2**	**Sub1**	0.56±0.05	0.65±0.01	0.33±0.03	0.71±0.08
	**Sub2**	0.23±0.02	0.20±0.00	0.16±0.01	0.22±0.01
	**Sub3**	0.01±0.00	0.02±0.00	0.01±0.00	0.02±0.01
	**Sub4**	0.37±0.07	0.20±0.00	0.18±0.02	0.66±0.16
	**Sub5**	0.33±0.10	0.42±0.04	0.22±0.02	0.48±0.07
	**Sub6**	0.87±0.01	0.90±0.01	0.78±0.01	0.91±0.04
	**Mean**	0.40 ± 0.02	0.40±0.01	0.28±0.01	0.50±0.03

**Table 4:** The average of AUC results for novelty detection using normative modeling, reconstruction-based and one-class-SVM trained only on the two available normal subjects (Subjects 4 and 10) of the FOG dataset.

Dataset	Subject	Normative	Reconstruction	1C-SVM
**FOG**	**Sub1**	0.81±0.01	0.63±0.00	0.76±0.00
	**Sub2**	0.92±0.00	0.81±0.00	0.65±0.02
	**Sub3**	0.75±0.08	0.67±0.01	0.41±0.05
	**Sub5**	0.82±0.01	0.69±0.00	0.63±0.02
	**Sub6**	0.51±0.05	0.41±0.01	0.77±0.03
	**Sub7**	0.67±0.03	0.53±0.00	0.65±0.01
	**Sub8**	0.41±0.03	0.41±0.01	0.71±0.03
	**Sub9**	0.58±0.09	0.44±0.01	0.67±0.04
	**Mean**	0.68 ± 0.02	0.57±0.00	0.65±0.01

## Abbreviations

The following abbreviations are used in this manuscript:

IMU	Inertial Measurement Unit
PD	Parkinson’s Disease
ASD	Autism Spectrum Disorder
FOG	Freezing Of Gait
SMM	Stereotypical Motor Movements
DAE	Denoising Autoencoder
GEVD	Generalized Extreme Value Distribution
CNN	Convolutional Neural Network
ReLU	Rectified Linear Units
SVM	Support Vector Machine
RBF	Radial Basis Function
ROC	Receiver Operating Characteristic
AUC	Area Under the Curve
AUPR	Area Under the Precision-Recall Curve
MC	Monte Carlo
MTGPR	Multi-Task Gaussian Process Regression

## Appendix A. Generalized Extreme Value Distribution

For a random variable x∈R, the cumulative distribution function of the GEVD, i.e., F(x), is defined as below [46]:

(A1) $$\begin{array}{c} F\left(x\right)=\left\{\begin{array}{cc} e^{-{\left[1+\xi \left(x-\mu \right)/\sigma \right]}^{-1/\xi }}, & \xi \neq 0\\ e^{-e^{\left[-(x-\mu )/\sigma \right]}}, & \xi =0 \end{array}\right., \end{array}$$

μ∈R and σ>0 are the location and scale parameter, respectively. ξ∈R is the shape parameter and depending on whether ξ<0, ξ=0 or ξ>0 the distribution follows the special cases of GEVD, namely Weibull, Gumbel and Fréchet, respectively.
